# Examination of teachers’ emotional intelligence competence perception levels in terms of sportive activity and different variables

**DOI:** 10.3389/fpsyg.2025.1613193

**Published:** 2025-07-28

**Authors:** Burak Tozoğlu, Sertaç Erciş

**Affiliations:** ^1^Erzurum Provincial Directorate of Youth Services and Sports, Erzurum, Türkiye; ^2^Department of Physical Education and Sports, Faculty of Sports Sciences, Atatürk University, Erzurum, Türkiye

**Keywords:** teacher, sport, sport psychology, activity, emotional intelligence

## Abstract

**Introduction:**

This study examines whether teachers’ emotional intelligence (EI) competence perception levels specifically dimensions such as self-regulation, empathy, and social skills—differ according to sports activity and demographic variables. EI plays a crucial role in fostering effective classroom management and teacher-student interaction. It was hypothesized that teachers who engage in both individual and team sports would demonstrate higher levels of emotional intelligence compared to those who do not participate in sports or engage in only one type.

**Method:**

Using a descriptive survey design, data were gathered from 422 secondary school teachers in Erzurum (204 female, 218 male) during the 2023–2024 academic year via the Personal Information Form and the Emotional Intelligence Trait Scale–Short Form (EITS-SF). Statistical analyses included t-tests, ANOVA (with η^2^), and Pearson correlation.

**Results:**

EI levels did not significantly differ by gender (*p* = 0.215), age (*p* = 0.737), service length (*p* = 0.511), or overall sport participation (*p* = 0.641). However, participants involved in both individual and team sports reported significantly higher EI scores (*M* = 88.96, SD = 17.90; *p* < 0.001, η^2^ = 0.06).

**Conclusion:**

Combining individual and team sports may enhance emotional intelligence more than participation in a single type or no activity. Future research should explore the mechanisms behind this interaction and its educational implications.

## 1 Introduction

The primary goal of education is to shape individuals who contribute meaningfully to society as responsible and effective members (Bhagat et al., 2021). This developmental process encompasses balanced growth in cognitive, emotional, physical, and social domains, while fostering critical thinking and empathy as essential life skills (Aydin and Gürdoğan, 2023). In addition, education plays a pivotal role in enhancing learners’ self-awareness, helping them explore their potential and make informed decisions about their future goals (Ceylan and Saygılı, 2022). It is posited that such educational outcomes can be achieved primarily through the active engagement of school administrators and teachers, who play a pivotal role in the educational process.

Recent studies highlight that teachers play a vital role not only in delivering academic content but also in guiding students through key stages of personal development and career orientation. Particularly in mentorship contexts, teachers significantly contribute to the development of professional awareness, classroom behavior management, and the formation of teaching identity in future educators (Huot et al., 2024).

It is thought that the ability of teachers to establish healthy relationships with their students is related to their ability to know their students well, understand them emotionally and control their own emotions. For this reason, the need for teachers to have high emotional intelligence competence comes to the fore. Teachers who possess strong emotional intelligence are more capable of building meaningful and supportive relationships with their students. Developing skills such as emotional awareness, self-regulation, and empathy enables educators to respond more effectively to students’ needs, manage classroom dynamics more positively, and reduce conflicts through improved communication and understanding (Pattiasina et al., 2024). For example, Rahman et al. (2024) demonstrated that teachers’ self-awareness, self-regulation, empathy, and social skills significantly predicted student motivation for academic learning. Moreover, a study by Chu et al. (2023) found that emotional intelligence played a mediating role in the relationship between teacher–student rapport and reduced emotional exhaustion among EFL students.

The concept of emotional intelligence was first introduced by Salovey and Mayer (1990). It refers to a person’s ability to recognize and understand both their own emotions and those of others, and to use this emotional insight to guide their thinking and behavior. In educational settings, emotional intelligence plays a critical role in helping teachers establish healthy communication with students and maintain emotional balance in the classroom environment. Emotional intelligence is a system that regulates a person’s emotions, and it is the ability of an individual to be aware of his/her own emotions and then the emotions of other individuals. Within the framework of social–emotional intelligence, individuals first go through a process of recognizing and expressing their own emotions. As they learn to regulate these emotions and become more attuned to the emotional states of others, they gain the ability to intentionally guide their thoughts and behaviors. This emotional competence fuels motivation and provides the energy needed for effective learning, communication, and social engagement (Arıg and Alcı, 2023). Individuals with a high level of emotional intelligence possess the capacity to utilize their emotions intentionally and effectively across various domains of life. By effectively managing emotional challenges encountered across professional, academic, and social settings, individuals can improve job performance, attain educational success, and cultivate healthier interpersonal relationships. Emotional intelligence, therefore, functions as a crucial capacity for navigating complex emotional experiences throughout various domains of life (Miao et al., 2022).

However, existing research provides limited insight into how lifestyle-related variables, such as participation in sports, might influence teachers’ perceived emotional intelligence. It is assumed that engaging in sports—particularly activities that promote teamwork, self-regulation, and empathy—may have a positive impact on emotional development. Supporting this, a 2024 study among Chinese university students showed that emotional intelligence exerted a direct influence on physical activity engagement, while achievement motivation partially mediated this relationship—highlighting the link between motivational and emotional factors and sporting activity levels (Kaya and Kılıç, 2022).

Sports activities play a multifaceted role in the development of individual personality and the process of socialization. Through participation in sports, individuals have the opportunity to experience and internalize social norms and values, while also enhancing key social skills such as teamwork, leadership, responsibility, and empathy. Involvement in physical activity contributes to psychological resilience, strengthens social connections, and positively shapes one’s interaction with the broader community. In this respect, sports serve as a comprehensive tool that supports personal growth and social integration simultaneously (Öztürk Karataş et al., 2021). Sports activities play a significant role in helping individuals maintain emotional and mental balance. During physical exercise, the release of hormones such as endorphins, dopamine, and melatonin contributes to a sense of relaxation and helps reduce stress levels. These biological effects allow individuals to manage emotions such as anxiety, anger, and aggression more effectively, promoting a greater sense of inner peace and supporting overall psychological well-being (Yeşilay, 2024).

In light of this, the current study aims to explore whether teachers’ perceived emotional intelligence competence differs according to their engagement in sports and key demographic variables such as gender, age, and teaching experience. Based on existing literature, it is hypothesized that teachers who regularly engage in both individual and team sports will report higher levels of emotional intelligence competence compared to those who do not participate in sports or engage in only one type of sport. Additionally, emotional intelligence levels are expected to vary by gender and teaching experience, with female and more experienced teachers demonstrating higher perceived emotional competence.

## 2 Methods

### 2.1 Research method

The present study aims to explore whether teachers’ perceived emotional intelligence (EI) competence levels vary based on demographic variables such as gender, age, and professional experience, as well as their level and type of engagement in sports activities. In line with this objective, a descriptive survey design was employed to reflect the existing state without any intervention. Additionally, correlational methods were used to examine the nature and strength of the relationships between EI and selected independent variables. This approach enables a broader understanding of how both personal and activity-related factors may be associated with teachers’ emotional competencies. Similar research published in 2021 utilized a descriptive–correlational design with convenience sampling among pre-service and in-service teachers and found meaningful associations between EI and demographic variables, providing a solid methodological parallel to the present study (Khademi and Rahmani, 2021).

### 2.2 Ethical committee approval

An ethics committee approval decision dated 21.03.2025 and numbered E-70400699-000-2500104962 was received for the scientific study.

### 2.3 Population and sample

This study was conducted with teachers working in public schools affiliated with the Ministry of National Education in Erzurum during the 2023–2024 academic year. The sample consisted of 422 teachers who voluntarily participated in the research, including 204 women and 218 men.

A convenience sampling method was used to determine the participants of the study. This non-probability sampling technique involves selecting individuals who are most readily accessible to the researcher. It enables data collection in a practical, fast, and cost-effective manner. Although this approach is efficient under time and logistical constraints, the findings may be limited in terms of generalizability due to the nature of the selected group. Recent literature also indicates that convenience sampling introduces multiple forms of bias—including selection bias and limited external validity and its results are only generalizable to the sampled group rather than the broader population (Golzar and Tajik, 2022).

### 2.4 Data collection tools and techniques

The data were collected using a two-part questionnaire administered face-to-face. In the first part, the “Personal Information Form” was used, and in the second part, the “Emotional Intelligence Trait Scale–Short Form (EITS-SF)” adapted to Turkish was applied (Deniz et al., 2013a). A recent study by Orhan (2024) examined the reliability of the Trait Emotional Intelligence Questionnaire–Short Form (TEIQue–SF) by analyzing a large number of previous findings. The results showed that the scale provides consistent and reliable outcomes across various age groups and demographic profiles. These findings suggest that the TEIQue–SF is a suitable tool for use in a wide range of populations (Orhan, 2024).

#### 2.4.1 Personal information form (PIF)

A five-item Personal Information Form was designed to collect demographic information from teachers, including gender, age, years of service, engagement in sports activities, and the type of sports they practiced. The selection of these variables was guided by commonly used demographic indicators in behavioral and educational research. The structure of the form aligns with the framework proposed by Fraenkel et al. (2019), who emphasize the role of demographic control variables in enhancing the interpretability of statistical results in social science studies. This approach ensures that individual differences potentially influencing emotional intelligence levels are accounted for at the data collection stage, as demonstrated in recent empirical studies employing the Personal Information Form (Kaya and Kılıç, 2022).

#### 2.4.2 Emotional intelligence traits scale–short form (EITS-SF)

The Emotional Intelligence Trait Scale–Short Form (EITS-SF), developed by Petrides and Furnham (2001) and adapted to Turkish by Deniz et al. (2013b), consists of 20 items rated on a 7-point Likert scale. High scores indicate high perceived emotional competence; low scores indicate the opposite. The Cronbach alpha coefficient of the EITS-SF was reported as 0.81 in these previous studies (Petrides and Furnham, 2001; Deniz et al., 2013b). The internal consistency coefficient for this study is presented in Table 1. To the best of our knowledge, no peer-reviewed research utilizing the EITS-SF has been published in the last 5 years.

**Table 1:** Reliability Cronbach’s Alpha value of emotional intelligence scale.

	Cronbach’s Alpha	*N* of items
Emotional intelligence	0.72	20

### 2.5 Statistical analysis

Before proceeding with statistical analyses, researchers must provide objective evidence that essential assumptions such as normality, linearity, homogeneity, and stationarity are met, as violations of these assumptions can significantly compromise the validity and interpretability of the results (Kurt and Doğan, 2021).

Recent studies emphasize the importance of checking key assumptions such as normality, linearity, homogeneity, and stationarity before conducting statistical analyses. If any violations are detected, researchers are encouraged to apply corrective methods or use robust or non-parametric techniques to ensure the reliability of their results (Brysbaert et al., 2023).

The research data were analyzed using SPSS version 22.00, a statistical software package released by IBM in 2013. Although this version is not the most recent, it continues to be widely used in educational and social science research. Recent studies published after 2021 have also reported using SPSS 22 for similar analyses (Kesmez, 2021; Demir et al., 2022).

The normality of Emotional Intelligence scores was assessed through skewness and kurtosis. The values fell within the ±1.50 threshold, indicating a normal distribution suitable for parametric tests. This threshold is widely recognized in the literature. Kesmez (2021) highlighted that the ±1.50 range proposed by Tabachnick and Fidell remains an accepted criterion in educational research. Demir et al. (2022) noted that skewness within ±2 and kurtosis within ±7 are also acceptable in large samples.

Accordingly, parametric tests were employed in the analysis. Independent Samples t-tests and One-Way ANOVA were used to assess group differences. Pearson correlation analysis was performed to examine relationships between emotional intelligence scores and continuous variables such as age and length of service. The level of statistical significance was set at *p* < 0.050.

## 3 Results

In this section, the tables and comments resulting from the statistical analyses conducted for the purpose of the research on the data obtained are presented.

The reliability analysis of the emotional intelligence scale used in the study revealed a Cronbach’s Alpha coefficient of 0.72, indicating an acceptable level of internal consistency (Table 1).

The descriptive statistics for the overall emotional intelligence scores were calculated and showed a mean of 80.24 with a standard deviation of 12.92 (Table 2).

**Table 2:** Descriptive statistics of the emotional intelligence scale.

*N*	422
Mean	80.24
Median	79.00
Mode	80.00
Std. Deviation	12.92
*Skewness*	***0.765***
*Kurtosis*	***1.574***

A total of 422 teachers, 204 female and 218 male, participated in the study. 207 of the teachers stated that they were doing sports activities and 215 of them stated that they were not. Of the teachers who were doing sports activities, 148 stated that they were doing individual sports, 32 stated that they were doing team sports and 27 stated that they were doing both types of sports (Table 3).

**Table 3:** Personal characteristics of participants.

Variable		*N*	%
Gender	Male	204	48.3
	Female	218	51.7
	Total	422	100.0
Sports activity status	Yes	207	49.1
	No	215	50.9
Type of sports activity he/she is doing	Individual	148	71.5
	Team	32	15.5
	Both	27	13.0

It was determined that the average age of the teachers participating in the study was 36.29 ± 9.24 and their average service period was 11.45 ± 8.27 (Table 4).

**Table 4:** Average age and service length of participants.

	Age	Years of service
*N*	422	422
Mean	36.29	11.45
Median	35.50	10.00
Mode	28.00	1.00
Std. Deviation	9.24	8.27

As a result of the t-test analysis conducted to determine the difference between male and female teachers in terms of teachers’ emotional intelligence competency perception levels, it was found that there was no difference at the *p* < 0.050 significance level (*p*; 0.215 > 0.050) (Table 5).

**Table 5:** Comparison of emotional intelligence competence perception levels according to gender.

Gender	*N*	x̅	Sd	*t*	*p*
Female	204	81.04	12.83	1.242	0.215
Man	218	79.48	12.99		

As a result of the t-test analysis conducted to determine the difference between teachers’ emotional intelligence competence perception levels according to their sports activity status, it was found that there was no difference at the significance level of *p* < 0.050 (*p*; 0.641 > 0.050) (Table 6).

**Table 6:** Comparison of emotional intelligence competence perception levels according to sporting activity status.

Sporting activity status	*N*	x̅	Sd	*t*	*p*
Yes	207	80.54	13.35	0.466	0.641
No	215	79.95	12.52		

As a result of the one-way analysis of variance (ANOVA) test conducted to determine the differentiation among teachers in terms of the type of sports activity they do in terms of their emotional intelligence competence perception levels, it was found that there was a significant difference at the *p* < 0.050 significance level (*p*; 0.001, 0.038, 0.003 < 0.050) (Table 7). It was found that the emotional intelligence competence perception levels of teachers who do both types of sports together are higher than those of teachers who do individual, team sports or do not do sports activities.

**Table 7:** Comparison of emotional intelligence competence perception levels according to the type of sporting activity they do.

Type of sporting activity	*N*	x̅	SD	*f*	*p*	Difference
Individual	148	79.11	12.00	4.637		
Team	32	80.03	12.69		0.001	3 > 1
Both	27	88.96	17.90		0.038	3 > 2
Do not	215	79.95	12.52		0.003	3 > 4
Total	422	80.24	12.92			

When the correlation analysis results were examined to determine the relationship between the age and length of service of teachers and their emotional intelligence competence perception levels, it was determined that there was no significant relationship between their age and length of service and their emotional intelligence competence perception levels at the *p* < 0.050 significance level (*p*; 0.737, 0.511 > 0.050) (Table 8).

**Table 8:** Correlation analysis results between participant’s age and years of service and emotional intelligence competency perception levels.

		Emotional intelligence competency perception levels
Age	Pearson Correlation	0.016
	Sig. (2-tailed)	0.737
Years of service	Pearson Correlation	0.032
	Sig. (2-tailed)	0.511

## 4 Discussion

A total of 422 teachers (204 female, 218 male) participated in the study conducted during the 2023–2024 academic year to examine differences in emotional intelligence competence perceptions based on sports activity and demographic variables. While the sample size enhances statistical power, limitations exist in representativeness due to the absence of data on participants’ geographic distribution and teaching branches. In addition, the homogeneity of variables such as age balance and years of service in the sample was not explained. The descriptive statistics in Table 4 indicate that the average age of the participants was 36.29 (SD = 9.24) and the average length of service was 11.45 years (SD = 8.27). The relatively high standard deviations suggest a wide distribution across both variables, implying a heterogeneous sample in terms of age and professional experience. This variability strengthens the study’s external validity by capturing a broader range of professional profiles, although it may introduce additional noise into group comparisons.

It was determined that there was no difference between male and female teachers in terms of their emotional intelligence competence perception levels at a significance level of *p* < 0.050. In their study titled “The relationship between emotional intelligence and organizational cynicism levels of high school teachers,” Erdem and İpek (2021) found that there was no difference in the emotional intelligence levels of female and male teachers. In her study on prospective teachers, Canpolat (2021) found that there was no significant difference in terms of emotional intelligence of prospective teachers according to their gender. In their study on athletes from different sports branches, Arslan and Kılıç (2021) found that there was no significant difference between gender and emotional intelligence and its sub-dimensions in the evaluation of emotional intelligence perception. In their study on gender differences in emotional intelligence. Recent studies have reported significant gender-based differences in emotional intelligence levels. For example, Cabello et al. (2021) found that female university athletes scored higher in emotional awareness, while male athletes demonstrated greater emotional control and regulation. Similarly, a study by Marôco et al. (2023) revealed that gender played a significant role in shaping emotional intelligence components, with social and cultural factors such as gender norms contributing to these differences. These findings align with and help explain the mixed results observed in previous studies, underscoring the importance of considering contextual variables—such as cultural norms and regional diversity—when interpreting gender-related emotional intelligence outcomes., Studies in the literature show both parallels and contrasts with the results of our study. This section effectively contextualizes the findings by relating them to previous research, which strengthens the academic value of the discussion. However, the interpretation of conflicting results regarding the effect of gender on emotional intelligence could be further enriched by considering explanatory variables such as cultural norms, regional differences, or diversity within the sample. These contextual factors might help explain the variation in findings across different studies and provide a more nuanced understanding of gender-related patterns in emotional intelligence.

It has been determined that there is no difference in the perception levels of emotional intelligence competence among teachers according to their sports activity status. Recent research also supports the absence of significant differences in emotional intelligence based on sports participation. For instance, Yıldız and Aydoğmuş (2023), in their study on university students, found no meaningful variation in emotional intelligence scores between those who participated in sports and those who did not. Similarly, Genç et al. (2022) reported that students who engaged in regular physical activity did not differ significantly in emotional intelligence compared to those who were inactive. Tuncel and Kardaş (2022) also observed no significant gender-based differences in emotional intelligence scores when comparing individuals with and without regular sports participation. In studies conducted by Elmas et al. (2021) on university students, Von Bothmer and Fridlund (2005) on Swiss university students, and Karaca on adults, it was determined that the level of physical activity did not differ according to gender. Studies in the literature present both similarities and differences when compared with the current study. For instance, recent research published in peer-reviewed journals has reported significant variations in multiple intelligence types among students who actively participate in sports versus those who do not.

Additionally, a study by Zayed et al. (2023) comparing sport practitioners and non-practitioners revealed that individuals engaged in regular physical activity demonstrated significantly higher levels of bodily–kinesthetic and emotional intelligences, suggesting that sports participation may contribute to the development of specific intelligence domains. Here, the consistency of the findings with the literature is shown in detail, which increases the credibility of the study. However, the concept of ‘sportive activity’ was not clarified. Factors such as the duration, type and intensity of the sports practiced were not specified, which limits the interpretation of the results of the study. Furthermore, beyond clarifying the definition and scope of sportive activity, it is also important to consider the potential influence of demographic variables on sport preferences. Factors such as gender, age, and teaching experience may shape whether individuals tend to engage in individual or team sports. Recent studies demonstrate that demographic characteristics such as gender and age significantly influence individuals’ sport type preferences and related psychological outcomes. For example, a 2024 study conducted with elite individual athletes found that emotional intelligence levels differed significantly across variables such as age, gender, and sport branch highlighting the nuanced role these demographic factors play in both sport engagement and emotional development (Özkara and Atalay, 2024). For instance, younger participants might favor team-based activities for their social aspects, whereas older or more experienced teachers may prefer individual sports that offer flexibility and autonomy. Although this study did not directly examine these associations, acknowledging them could provide a deeper understanding of the interplay between lifestyle choices and emotional intelligence development. Future research should aim to address this gap to enrich the interpretive framework surrounding sport-related variables. In interpreting the findings, it is important to note that the definition of “sport participation” in this study was based on a binary self-report item (“Do you engage in sports activities?”). The study did not collect data on frequency, duration, or intensity of these activities, which may have influenced the sensitivity of the comparisons. Future studies would benefit from using standardized physical activity inventories to capture more nuanced information regarding the nature of sports involvement.

It has been determined that there is a significant difference in the level of emotional intelligence competence perception of teachers according to the type of sporting activity they do. It was found that the emotional intelligence competence perception levels of teachers who do both types of sports together are higher than those of teachers who do individual, team sports or do not do sports activities. One possible explanation for the higher emotional intelligence scores among participants engaged in both individual and team sports lies in the complementary skills fostered by each format. Individual sports may cultivate self-regulation, goal-setting, and internal motivation, while team sports encourage collaboration, empathy, and communication. Engaging in both types of activities may promote a more holistic development of emotional competence by stimulating diverse emotional and social experiences. In their study on elite athletes, Salman et al. (2018) found that there was a significant difference between team and individual athletes in terms of the empathy and social skills sub-dimensions of emotional intelligence in national athletes in individual and team sports. Sezer and Tutal, in their peer-reviewed study, examined emotional intelligence levels among university students involved in both individual and team sports. Their findings revealed that students who engaged in team sports scored significantly higher in the emotional intelligence sub-dimensions of optimism and mood regulation. This supports the view that participation in collaborative physical activities positively contributes to emotional development (Tuncel and Kardaş, 2022). In their article on licensed athletes, Rodriguez-Romo et al. found that there was a significant difference between the athletes according to the type of sport they were doing (Rodriguez-Romo et al., 2021). Studies in the literature are similar to the results of our study.

This finding is one of the unique aspects of the study because not only the type of sport, but also the type of sport may affect emotional intelligence. Such detailed analyses would enrich the study. However, the number of sport types and the size of these groups were not specified; this may affect the reliability of statistical interpretation. In the present study, of the 207 teachers who reported engaging in sports, 148 (71.5%) stated they practiced individual sports, 32 (15.5%) team sports, and 27 (13%) both types. These subgroup sizes reveal a clear imbalance between groups, particularly with the “both” category having the smallest number of participants. This unequal distribution could potentially influence statistical reliability and limit generalizability. Therefore, future studies may benefit from ensuring more balanced group sizes or applying statistical corrections such as bootstrapping or weighting methods to validate subgroup comparisons more robustly.

It was determined that there was no significant relationship between the age and length of service of the teachers and their emotional intelligence competence perception levels. In their study, Gaitniece-Putāne and Raščevska (2006) found that the interaction of age and gender did not have a significant effect on emotional intelligence measured by five separate factors. Sevindik et al. (2012) observed in their study on students that there was no significant difference in emotional intelligence levels between age groups. In their study with high school teachers, Erdem and Ipek (2021) found no difference in the total emotional intelligence scores between different age groups and professional tenure. Conversely, Toytok (2013) found a significant difference in all dimensions of emotional intelligence according to professional seniority; teachers with higher tenure reported higher EI across sub-dimensions. Pasand (2021) found a significant relationship between emotional intelligence and age variables, specifically in the sub-dimensions of problem solving, independent action, realism, interpersonal relations, responsibility, and empathy. In their study with pre-school teachers, Ertuğrul and Kutluca (2020) found no significant difference in EI levels in terms of age and length of service variables. Similarly, in their study with healthcare professionals, Yılmaz and Güler (2022) found no significant difference between total emotional intelligence scores and professional seniority. Recent findings by Peacock et al. (2024) indicate that there were no statistically significant differences in nurses’ overall emotional intelligence scores when analyzed by age, educational attainment, or professional experience. This suggests that emotional intelligence levels among nurses remain consistent regardless of demographic or tenure-based variables. A study conducted by Chan and Pyland (2022) among undergraduate students revealed that age was positively and significantly correlated with various emotional intelligence subdimensions, including problem-solving, empathy, interpersonal relationships, self-regulation, and reality-testing. These findings suggest that emotional competencies in these domains tend to improve with increasing age. A recent study by Reddy and Nair (2022) examined emotional intelligence levels among university students across different age groups and found no statistically significant differences. These findings suggest that age may not play a decisive role in shaping emotional intelligence during the university years, indicating a relative stability of emotional competencies regardless of students’ age. Studies in the literature show similarities and differences with the results of our study.

This finding suggests that emotional intelligence may be shaped not only by age or length of service but also by more subjective factors such as personality traits, emotional awareness training and quality of social interaction. Although the current study primarily focused on how emotional intelligence varies by sports activity and demographic variables, it did not analyze whether demographic characteristics influence sport type preferences. Future research could benefit from examining whether factors such as gender, age, and teaching experience are associated with a greater likelihood of participating in individual versus team-based sports. Such insights would offer a more nuanced understanding of how personal traits and lifestyle choices intersect in shaping emotional competence.

Since the data used in the study were obtained through self-report, social favorability effect may have occurred in the participants’ responses. In addition, emotional intelligence was assessed only in terms of perception, and behavioral observations or third-party assessments were not included. Measurements of sportive activity were also subjective and not supported by objective data such as duration and intensity. These limitations are important in terms of guiding future studies.

This study provides high external validity with its large sample size (n = 422). At the same time, analyzing sports not only according to whether they are practiced or not, but also according to their type makes this study one of the rare studies that contribute to the gaps in the literature. The fact that it comprehensively addresses the effects of different demographic variables on emotional intelligence provides important information at the intersection of education and sport psychology. Future research should consider incorporating third-party assessments and objective measures of sports engagement (e.g., frequency, duration, and intensity). Including variables such as school type, training background, or psychological well-being could also enhance explanatory power. Moreover, the findings suggest that integrating emotional intelligence training into teacher development programs may offer practical benefits.

## 5 Conclusion

In conclusion, while demographic variables and overall sports participation did not significantly influence emotional intelligence (EI), sport type emerged as a differentiating factor. Teachers engaged in both individual and team sports reported higher EI levels. These findings suggest that varied physical activity may support emotional development. These results partially confirmed the initial hypothesis, supporting the expectation that teachers involved in both individual and team sports would display higher emotional intelligence levels, while demographic factors showed no significant effect. Future studies should explore this relationship using broader and longitudinal samples.

## Data Availability

The raw data supporting the conclusions of this article will be made available by the authors, without undue reservation.
